# Satisfaction with access to health services among foreign-born population in Finland: a survey-based study

**DOI:** 10.1186/s12913-022-08155-3

**Published:** 2022-06-15

**Authors:** Valentina Kieseppä, Regina García Velázquez, Tuulikki Vehko, Hannamaria Kuusio

**Affiliations:** Finnish Institute for Health and Welfare, Public Health and Welfare, P.O. Box 30, 00271 Helsinki, Finland

**Keywords:** Emigrants and immigrants, Access to health care, Health services, Survey

## Abstract

**Background:**

Many European studies have shown migrants to be less satisfied with health care and find it less accessible than the general populations. The aim of this study was to compare satisfaction with access to health care between migrants from different regions of origin and the general population of Finland.

**Methods:**

This study uses data from two comprehensive survey samples on health and wellbeing of the foreign-born and the general population living in Finland. Three aspects of satisfaction with health care access were measured and predicted by region of origin using logistic regression.

**Results:**

Foreign-born population was slightly more dissatisfied with all aspects of the access to health care as compared to the general population. In all aspects of access, migrants from the Middle East and Africa were least likely to be satisfied.

**Conclusions:**

As the satisfaction with access was lowest among migrant groups which are likely to have higher needs for at least some health services in comparison to the general population, these results are alarming. More research is needed to identify the potential development points in the health care system of Finland.

**Supplementary Information:**

The online version contains supplementary material available at 10.1186/s12913-022-08155-3.

## Background

Equal access to health care is important in ensuring timely and appropriate treatment. There are many studies showing that migrants are generally less satisfied with health care [1–4] and find it less accessible [3, 5, 6] than general populations.

In 2020, there were nearly 421 000 (7.6% of the total population) foreign-born individuals in Finland [7], and this figure has been steadily increasing during recent years. In Finland, the largest migrant groups are migrants from the former Soviet Union or Russia, Estonia and Sweden, but migrants from Iraq, China, Somalia and Thailand also make up a large proportion of the foreign-born population [7]. As migrants form a very heterogeneous population, the health care needs of different migrant groups are likely to vary. It is increasingly important that the health care system can meet the needs of the changing population of Finland.

In Finland, there are three different health care systems: public health care, which is relatively affordable for everyone, private health care, and occupational health care. There are differences in waiting times, user fees and scope between the different systems. Typically, employed people can choose between these above-mentioned health service systems, whereas for low-income unemployed people the public health care is usually the only option [8].

Migration is in itself a very challenging experience, but the majority of migrating individuals are young and healthy, which makes the association between migration and health complicated: paradoxically, in many developed countries it has been documented that migrants are, in fact, healthier than native populations [9]. In Finland, people of foreign-born background generally report slightly worse health than the general population, although despite this, they report using slightly less health services than the general population [10]. This discrepancy might reflect barriers to care.

It is difficult to compare the studies on satisfaction with health care, since they have focused on different types of services, used different measures for satisfaction and perceived access, and as the health care systems are different. However, migrants and ethnic minorities have been found to be less satisfied with health care at least with maternity care [3, 4, 11], emergency care [12, 13] and mental health care [14] in European, North American and Australian studies.

There are also differences in satisfaction with health care between different migrant groups. In Norway, it has been found that especially migrants from Turkey, Iran, Pakistan and Vietnam were dissatisfied with their visits to general practitioners [1], and dissatisfaction with rehabilitation services especially among migrants from Turkey has also been found in Germany [15]. In Denmark, it has been found that both patients and professionals in emergency care were less satisfied when the patients were of Middle Eastern origin [16].

In Finland, a previous study has investigated the satisfaction of Somali women with maternity health services [17]. While the women were generally satisfied with the services, the attitudes of the health care professionals were perceived as unfriendly, and there were also communicational problems. However, comprehensive studies of satisfaction with access to health care among migrants have not yet been conducted in Finland.

The aim of this study is to compare differences in the satisfaction with access to health care between migrants from different regions to that of the general population living in Finland. In this study, migrants were defined as individuals who were born outside of Finland and whose parents or the only known parent were born outside of Finland. We focus on satisfaction with three aspects of access: 1) contacting process with the place of care, 2) getting the appointment without undue delay, and 3) getting examined without undue delay. As satisfaction and access to health care have important implications for adherence and treatment, this information can provide important insights into potential barriers in the health care system of Finland.

## Methods

This study uses data from the Survey on Well-Being among Foreign Born Population (FinMonik) and the National FinSote Survey (FinSote) 2018. Both surveys are implemented by the Finnish Institute for the Health and Welfare (THL) and have been approved by the Institutional Review Board of THL (Finmonik: THL/271/6.02.01/2018, Finsote: THL/637/6.02.01/2017). Informed consent was obtained from all subjects involved in the study.

### The FinMonik sample

The FinMonik survey data contains information on the well-being of the foreign-born population living in Finland. The sample was attained from the population register (maintained by the Digital and Population Data Service Agency) in March 2018. The sample was based on a stratified random sampling, described in more detail elsewhere [18]. The following criteria were used to draw the sample: (1) the respondent’s country of birth must be other than Finland; (2) both parents or the only known parent of the respondent must have been born abroad; (3) the respondent must have lived in Finland for at least a year at the time of sampling; (4) the respondent must be aged 18–64 at the time of sampling, and (5) the respondent must not have come to Finland through adoption.

13 650 individuals fulfilling the criteria formed the sample and received the invitation letter to participate in the study. Those who received the invitation letter but had moved abroad after sampling or whose invitation letter were returned undelivered by the post were considered empty records or out-of-score units (5.7%; n = 773). The final sample consisted of 12 877 respondents, of whom 6 836 (53.1%) participated in the study. Of these, 6489 had answered to the full version of the questionnaire and were included in this study.

The data collection is described in detail by Kuusio et al. [18]. The data were collected between 7 March 2018 and 15 January 2019 primarily online with an electronic questionnaire, and it was supplemented with a paper questionnaire and telephone interviews and home visits in the city of Espoo. The invitation letter and the questionnaire were translated into 17 languages (from Finnish to Albanian, Arabic, Dari, Farsi, English, Spanish, Mandarin Chinese, Kurdish (Sorani), Polish, French, Swedish, Somali, Thai, Turkish, Russian, Vietnamese, and Estonian). 77.1% of those invited to the study received the material in their declared mother tongue.

### The FinSote sample

The FinSote survey is a national, annually carried out survey on health, well-being and service use of the general population living in Finland. The data from the FinSote survey from year 2018 is used as a comparison data here.

The sample was selected by random sampling from the Digital and Population Services Agency. The respondents were selected so that they are over 20 years old (no upper age limit) and permanently living in Finland. Information was collected by mail and electronic questionnaires (available in Finnish, Swedish, English, and Russian). The questionnaire was sent to 60 000 individuals. 26 422 individuals responded (response rate 45.3%). For this study, individuals aged over 65 years were removed so that the samples would be comparable (*n* = 11 378).

### Outcome variables

Both FinMonik and FinSote surveys measured the satisfaction with health services with the following items: *Think about your experiences of using health services in the past 12 months. How were the below aspects achieved in your case*: *1) I was able to contact the place of care smoothly, 2) I was able to make an appointment without undue delay,* and *3) I was examined without undue delay (eg. laboratory tests, X-ray, ultrasound)*. Each item had the following answer options: *1) always, 2) most of the time, 3) sometimes, 4) never, 5) does not apply to me (I have not used health services).* Those who had chosen the fifth category were excluded from the analyses. The three satisfaction items were dichotomized for the regression models: *satisfied* (categories *always* or *most of the time*) and dissatisfied (categories *sometimes* or *never*).

### Region of origin

Information on the *region of origin* was attained from the Digital and Population Services Agency. The country of origin was based on definition by Statistics Finland; country of origin was primarily defined as the mother’s country of birth, if that was unavailable, then as the father’s country of birth, if that was unavailable, then as the individual’s own country of birth. It was categorized as follows: 1) Russia, 2) Estonia, 3) Rest of Europe, North America and Oceania, 4) Middle East and North Africa, 5) Rest of Africa, 6) Southeast Asia, 7) East Asia, 8) South and Central Asia, 9) Latin America, 10) General population. The last category includes all the individuals from the FinSote survey data, and it should be noted that this category might also include a small number of foreign-born individuals. The categorization of the region of origins was based on the United Nations Standard country or area codes for statistical use [19], with some modifications for Finnish context (e.g., representing Russia and Estonia as their own groups). The composition of the groups by country of birth is described in detail elsewhere [20]. The “Russia” group also involves people whose country of origin is former Soviet Union.

### Covariates

Information on *sex* and *age* (categorized into the following categories: 18–29, 30–49 and 50–64) were attained from the Digital and Population Services Agency. Other covariates obtained from the questionnaires included *quality of life* (measured with the item: *How would you rate your quality of life?* 1) *very poor*, 2) *poor*, 3) *neither poor nor good*, 4) *good*, 5) *very good*) and chronic illness (measured with the item *Do you have a chronic illness or other chronic health problem*? 1) *yes* 2) *no*).

### Statistical analysis

Initially, we compared the distributions of answers to the outcome questions between the different region of origin groups, to make sure some groups did not systematically leave the questions unanswered more often than others. To make sure that there was no tendency for the region of origin -groups to respond systemically differently to the items differential item functioning (DIF) was measured. This was done by using the R’s *lordif* package [21]. We examined both uniform and non-uniform DIF. No DIF was detected for the items included in this study.

The two datasets were combined and transformed into a survey object (using R’s *survey* package [22]) using the appropriate survey weights. Participants with missing data on the variables of interest were excluded from the analyses. Separate dichotomous logistic regression models were calculated for each outcome variable. The region of origin was the main explanatory variable in all the three models and “General population” was chosen as the reference category. Both unadjusted and adjusted estimates were produced. The models were adjusted for sex, age, quality of life and chronic illness. To better visualize the results, the predicted marginal probabilities for being satisfied were calculated for each adjusted model. To assess the goodness-of-fit of the models, Nagelkerke R^2^ values were calculated for each adjusted model.

## Results

The descriptive characteristics of the samples are presented in Table 1 (absolute counts and weighted percentages). Both samples had nearly equal weighted proportions of men and women. Most participants were in the age group 30–49, but larger proportion of the general population sample were in the oldest age group (50–64). Most participants estimated their quality of life to be good. Higher weighted proportion of the general population than the foreign-born population reported having a chronic illness (43.6% vs. 31.6%), which might reflect the age differences between the samples.

**Table 1:** Descriptive characteristics of the samples (n, %) (absolute counts, sample-weighted percentages)

	Foreign-born	General population
Total (% of the total sample)	6489 (25.4)	11,378 (74.6)
Sex		
Female	3573 (48.0)	6465 (49.5)
Male	2916 (52.0)	4913 (50.5)
Age		
18–29	1408 (22.0)	1587 (20.9)
30–49	3569 (56.0)	3778 (43.0)
50–64	1512 (21.9)	6013 (36.1)
Quality of life		
Very poor	42 (1.2)	72 (0.9)
Poor	138 (2.5)	411 (3.8)
Neither poor nor good	1255 (20.6)	2055 (16.8)
Good	3736 (55.2)	6498 (56.1)
Very good	1137 (17.6)	2212 (20.8)
Did not answer	181 (3.0)	130 (1.5)
Chronic illness		
Yes	2157 (31.6)	5461 (43.6)
No	4123 (64.9)	5711 (54.5)
Did not answer	209 (3.5)	206 (1.9)
Region of origin		
Estonia	618 (13.6)	
Russia	1949 (21.9)	
Rest of Europe, North America and Oceania	1203 (19.1)	
Middle East and Northern Africa	962 (15.8)	
Rest of Africa	310 (9.1)	
Southeast Asia	684 (8.4)	
East Asia	301 (4.2)	
South and Central Asia	303 (4.6)	
Latin America	159 (3.3)	

Table 2 shows the distributions (absolute counts, sample-weighted percentages) of the responses to the questions measuring satisfaction with the service access between the samples. Foreign-born individuals were more likely to leave the questions unanswered, whereas individuals from the general population sample were more likely to report not to have used health services. The trends of responding between the two samples were generally similar. Individuals in both samples tended to be satisfied with treatment access. However, foreign-born individuals were more often less satisfied: 12.3% of the foreign-born sample responded that they were able to contact the place of care smoothly only “sometimes”, while the respective figure was 8.1% for the general population. 4.5% of the foreign-born population responded that they had “never” been able to make an appointment without delay, and 17.5% responded that they had only “sometimes” been able to make an appointment without delay (the respective figures were 2.9% and 13.1% for the general population). Similarly, 13.3% of the foreign-born sample responded that they were examined without undue delay “sometimes”, when the respective figure was 9% for the general population sample. The distributions of responses in different region of origin categories across the three questions are available in the supplementary material (supplementary Figs. 1–3).

**Table 2:** Distributions of responses to questions concerning the satisfaction with treatment access among the foreign-born and the general population samples (n, %) (absolute counts, sample-weighted percentages)

	Question 1		Question 2		Question 3	
Response	Foreign-born	General population	Foreign-born	General population	Foreign-born	General population
(1) Always	2282 (33.8)	3456 (32.2)	1813 (28.0)	3119 (28.3)	1987 (30.9)	3293 (28.8)
(2) Most of the time	1931 (29.0)	4275 (35.5)	1944 (27.9)	4289 (35.0)	1705 (24.7)	3623 (29.4)
(3) Sometimes	775 (12.3)	931 (8.1)	1130 (17.5)	1454 (13.1)	840 (13.3)	1072 (9.0)
(4) Never	154 (2.1)	153 (1.2)	290 (4.5)	297 (2.9)	286 (3.7)	206 (2.2)
(5) Does not apply to me	832 (13.9)	2114 (18.2)	799 (13.0)	1788 (15.9)	1118 (17.9)	2741 (25.6)
Unanswered	515 (8.8)	449 (4.8)	513 (9.0)	431 (4.8)	553 (9.4)	443 (4.9)

Question 1 = “I was able to contact the place of care smoothly”, Question 2 = “I was able to make an appointment without undue delay”, Question 3 = “I was examined without undue delay (eg. laboratory tests, X-ray, ultrasound)”

### Logistic regression analysis

The results of the unadjusted logistic regression models are presented in Table 3.

**Table 3:** Satisfaction with treatment access as predicted by region of origin (ref. General population). Unadjusted estimates

	Odds Ratios (ORs) (95% CI)		
	Contact smoothly	Appointment without delay	Examinations without delay
	(1)	(2)	(3)
Russia	0.798	0.844	0.744^**^
	(0.625, 1.020)	(0.684, 1.043)	(0.599, 0.925)
Estonia	1.318	1.271	0.772
	(0.863, 2.012)	(0.874, 1.847)	(0.535, 1.115)
Rest of Europe, North America, and Oceania	0.599^**^	0.624^***^	0.743
	(0.437, 0.820)	(0.488, 0.799)	(0.551, 1.002)
Middle East and North Africa	0.278^***^	0.309^***^	0.394^***^
	(0.208, 0.372)	(0.238, 0.400)	(0.294, 0.529)
Rest of Africa	0.347^***^	0.430^***^	0.390^***^
	(0.209, 0.576)	(0.275, 0.675)	(0.245, 0.621)
Southeast Asia	0.501^***^	0.639^**^	0.580^**^
	(0.364, 0.689)	(0.470, 0.870)	(0.415, 0.809)
East Asia	1.519	1.076	0.596
	(0.809, 2.852)	(0.568, 2.040)	(0.332, 1.069)
South and Central Asia	0.838	0.663	0.967
	(0.502, 1.397)	(0.439, 1.001)	(0.597, 1.565)
Latin America	2.465^*^	1.240	2.168^*^
	(1.194, 5.089)	(0.677, 2.272)	(1.062, 4.426)

This table was created by using the *stargazer* package in R [23]

^*^p < 0.05

^**^p < 0.01

^***^p < 0.001

The results of the adjusted logistic regression models are presented in the Table 4.

**Table 4:** Satisfaction with treatment access as predicted by region of origin (ref. General population). Adjusted estimates

	Adjusted Odds Ratios (aORs) (95% CI)		
	Contact smoothly	Appointment without delay	Examinations without delay
	(1)	(2)	(3)
Russia	0.792	0.839	0.779^*^
	(0.612, 1.024)	(0.673, 1.045)	(0.621, 0.976)
Estonia	1.290	1.343	0.842
	(0.851, 1.954)	(0.924, 1.953)	(0.581, 1.222)
Rest of Europe, North America and Oceania	0.584^***^	0.627^***^	0.807
	(0.428, 0.797)	(0.491, 0.801)	(0.590, 1.102)
Middle East and North Africa	0.315^***^	0.351^***^	0.469^***^
	(0.230, 0.430)	(0.260, 0.473)	(0.334, 0.659)
Rest of Africa	0.295^***^	0.364^***^	0.364^***^
	(0.167, 0.521)	(0.220, 0.600)	(0.218, 0.607)
Southeast Asia	0.542^***^	0.685^*^	0.669^*^
	(0.391, 0.752)	(0.496, 0.945)	(0.473, 0.946)
East Asia	2.191^*^	1.316	0.756
	(1.097, 4.379)	(0.667, 2.596)	(0.396, 1.445)
South and Central Asia	0.790	0.631^*^	1.012
	(0.467, 1.337)	(0.406, 0.979)	(0.597, 1.718)
Latin America	2.128^*^	1.076	1.965
	(1.014, 4.466)	(0.566, 2.049)	(0.944, 4.093)
Nagelkerke R^2^	0.098	0.098	0.076

Models are adjusted for sex, age, chronic illness and quality of life

This table was created by using the *stargazer* package in R [23]

^*^p < 0.05

^**^p < 0.01

^***^p < 0.001

### Contact with the place of care

**Fig. 1:**
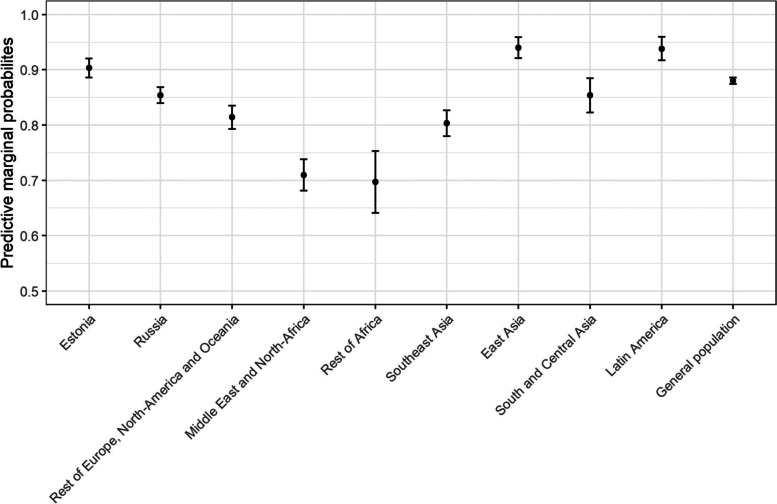
The predictive marginal probabilities and standard errors for satisfaction with contact with the place of care between migrants from different regions of origin and general population

The results of the first regression model predicting satisfaction with the contact with the place of care are further visualized in Fig. 1 as predicted marginal probabilities. Migrants from Western countries (excluding Estonia), the Middle East, Africa and Southeast Asia were less likely to be satisfied with the contact with the place of care as compared to the general population, whereas migrants from East Asia and Latin America were more likely to be satisfied with the place of care as compared to the general population.

Least likely to be satisfied with the contacting process were migrants from the Middle East and Northern Africa (aOR = 0.32, 95% CI = 0.23–0.43) and migrants from Sub Saharan Africa (aOR = 0.30, 95% CI = 0.17–0.52). Migrants from South East Asia were also less likely to be satisfied with the contact (aOR = 0.52, 95% CI = 0.39–0.75), whereas migrants from East Asia were more likely to be satisfied with the contact (aOR = 2.19, 95% CI = 1.10–4.38) than the general population.

### Appointment without delay

**Fig. 2:**
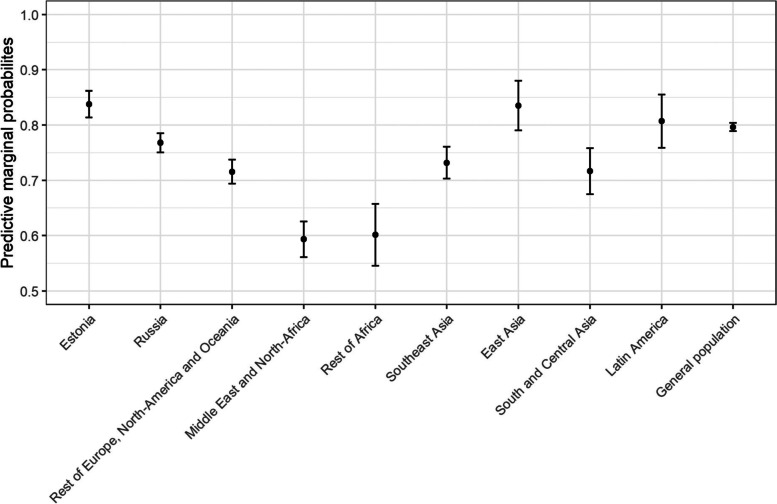
The predictive marginal probabilities and standard errors for satisfaction with getting the appointment without delay between migrants from different regions of origin and general population

The results of second regression model, predicting the satisfaction with getting the appointment without delay, are visualized in Fig. 2. Compared to the general population, migrants from Western countries (excluding Estonia), the Middle East, Africa, Southeast Asia and South and Central Asia were less likely to be satisfied with getting the appointment without delay. No migrant group was significantly more likely to be satisfied with getting the appointment without delay compared to the general population.

Least likely to be satisfied with getting the appointment were migrants from the Middle East and Northern Africa (aOR = 0.35, 95% CI = 0.26–0.47) and migrants from Sub Saharan Africa (aOR = 0.36, 95% CI = 0.22–0.60).

### Examinations without delay

**Fig. 3:**
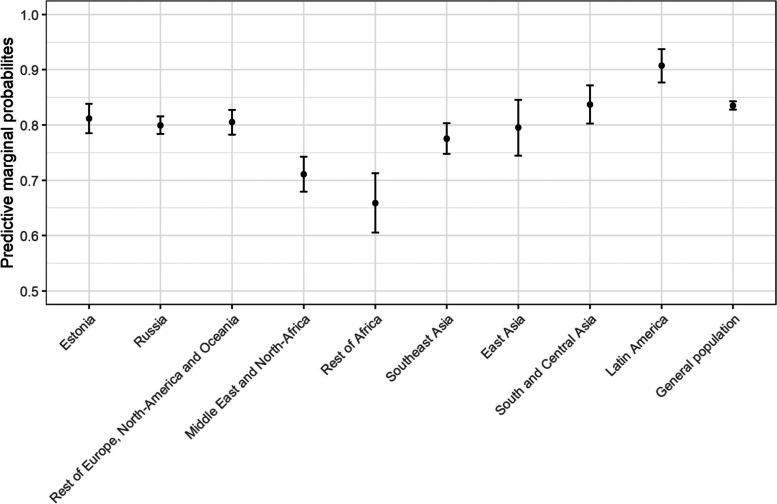
The predictive marginal probabilities and standard errors for satisfaction with getting the examinations without delay between migrants from different regions of origin and general population

The results of the third regression model, predicting satisfaction with getting examined without delay, are further visualized in Fig. 3. Compared to the general population, migrants from Russia, Middle East, Africa and Southeast Asia were less likely to be satisfied with getting examined without delay. No other group was significantly more likely to report being satisfied with getting examined compared to the general population.

The differences were most pronounced for migrants from Sub Saharan Africa (aOR = 0.36, 95% CI = 0.22- 0.61) and the Middle East and North Africa (aOR = 0.47, 95% CI = 0.33–0.66).

## Discussion

Generally, both the foreign-born population and the general population were satisfied with the access to the health services. However, while the trends of responding to the questions were similar between the samples, the foreign-born population was slightly more dissatisfied with all aspects of the access to care measured in this study.

There were differences in satisfaction between groups of different regions of origin. Migrants from Western countries (excluding Estonia), Middle East, Africa, Southeast Asia, South and Central Asia and Russia were less likely to be satisfied with at least one aspect of the health care access as compared to the general population. Migrants from the Middle East, Africa and Southeast Asia were less likely to be satisfied with all aspects of access compared to the general population. Migrants from East Asia and Latin America were more satisfied with the contacting process with the place of care as compared to the general population, but there were no significant differences for other aspects of access for these groups. In all aspects of access to health care, migrants from the Middle East and Africa were least likely to be satisfied as compared to the general population.

Many studies have found migrants to be in general less satisfied with health care as compared to general populations [1–4]. However, our study shows that there are important differences in satisfaction with access to health care between different migrant groups. In particular, the dissatisfaction with access among migrants from the Middle East and Africa is notable.

The dissatisfaction among these groups is alarming, as previous results from this same data have shown that migrants from Middle East and North Africa report lower self-perceived health and more diabetes, depression and other mental health symptoms as compared to the general population, and migrant women from Middle East and Africa have increased needs for maternity services [10]. Findings of lower self-perceived health among migrants of Kurdish origin have also been made in another Finnish study [24]. The high rates of mental health symptoms among migrants from Middle East and North Africa are particularly notable [25, 26].

As dissatisfaction was particularly notable in certain groups, it might reflect inequalities in the health care system. Possible inequalities in access to health care can worsen existing health inequalities.

There are different types of barriers migrants might face when attempting to access health care. Dissatisfaction with access to health care has been associated with inadequate knowledge of the health care system, health care staff acting as gatekeepers, and communicational barriers [27]. Long waiting times have also been identified as the reason for dissatisfaction among migrants [28, 29]. In Finland, barriers to health care among migrants of Russian and Kurdish origin were found to include long waiting times, high costs of treatment, communicational difficulties, uncertainness of how to access treatment, doubt over the helpfulness of treatment, mistrust of the skills of the professionals, and negative experiences of interactions with clinicians [24]. Overall, the permanent problem of Finnish primary health care is the poor access to treatment, due to long waiting times, particularly among unemployed, who do not have access to occupational health care. In addition, there are also regional differences in availability of services, particularly for primary care [30].

Based on previous findings, it could be expected that migrants from the Middle East and North Africa would have increased need especially for mental health services. In addition to the barriers mentioned above, there are some specific barriers which can complicate access to mental health services among people with different cultural backgrounds. They include stigma associated with mental disorders [31, 32], as well as different conceptions of what mental health issues are and what is appropriate treatment for them [33–35].

Many of these above-mentioned barriers can accumulate on individuals who are already in a vulnerable position, such as refugees. And indeed, in this study we found that migrants from refugee-generating countries had the highest odds for dissatisfaction. Our research results emphasize how important it is not to consider migrants as a uniform group in relation to health care, as different migrants can face different barriers, and for some, they can be more difficult to overcome than for others.

### Limitations

There are some limitations associated with this study. We did not have the information on what kind of health services the participants had used, so we do not know how much of the differences in satisfaction could be explained by the use of different kind of services. It is possible that, for example, the foreign-born population used more public health services whereas the general population used more of private services, which caused differences in satisfaction.

There are regional differences in access to health care, and since the two survey samples were not matched by region of residence, that might explain some of the disparities in satisfaction with access between the two populations. The two samples were also asymmetrical in relation to age: the general population was generally older, and had a lower age limit of 20, whereas the foreign-born sample included individuals aged at least 18.

Also, it has been shown that socioeconomic status is associated with satisfaction with health care [6]. The two survey samples compared in this study had measured socioeconomic status differently, so the models could not be adjusted with socioeconomic status, and the possible effect of socioeconomic status could not be controlled.

It should be noted a small part of the individuals in the general population sample might be foreign-born. Finally, because of the differences of health care systems across countries, generalization of the results should be done carefully.

## Conclusions

We found that migrants in general were less satisfied with access to health care as compared to the general Finnish population, although there were differences between different migrant groups, highlighting the fact that migrants should not be considered as a one, uniform group. Migrants from the Middle East and Africa were particularly likely to be dissatisfied with access. As migrants from these areas are likely to have higher needs for at least some health services, these results are alarming. These patterns may be due to the inability of the health service system to address the needs of people of different cultural backgrounds. Potential causes of dissatisfaction might include lack of knowledge of the services, language barriers, long waiting times, or negative experiences with the services. These barriers can accumulate on individuals who already are in a vulnerable position. More research is needed to study the explanations for the lower satisfaction among these groups, so that potential barriers in the health care system can be identified and interventions targeted correctly, to ensure appropriate and good quality care for people of all backgrounds.

## Supplementary Information

**Additional file 1: Supplementary figure 1.** The distribution of responses to the item"I was able to contact the place of care smoothly" between migrantsfrom different regions of origins and general population. **Supplementary figure 2**. The distribution of responses to the item"I was able to make an appointment without undue delay" betweenmigrants from different regions of origins and general population. **Supplementary figure 3.** Thedistribution of responses to the item "I was examined without undue delay”between migrants from different regions of origin and general population.

## Data Availability

The data presented in this study can be requested from the THL contacting the corresponding author.
