# Supplementary material for: Satisfaction with access to health services among foreign-born population in Finland: a survey-based study
Source: BMC Health Serv Res. 2022 Jun 15;22:781. doi: 10.1186/s12913-022-08155-3 (PMC9199131; doi:10.1186/s12913-022-08155-3)
Supplement: Supplementary file 1 — Additional file 1: Supplementary figure 1. The distribution of responses to the item"I was able to contact the place of care smoothly" between migrantsfrom different regions of origins and general population. Supplementary figure 2. The distribution of responses to the item"I was able to make an appointment without undue delay" betweenmigrants from different regions of origins and general population. Supplementary figure 3. Thedistribution of responses to the item "I was examined without undue delay”between migrants from different regions of origin and general population. [file 12913_2022_8155_MOESM1_ESM.docx]

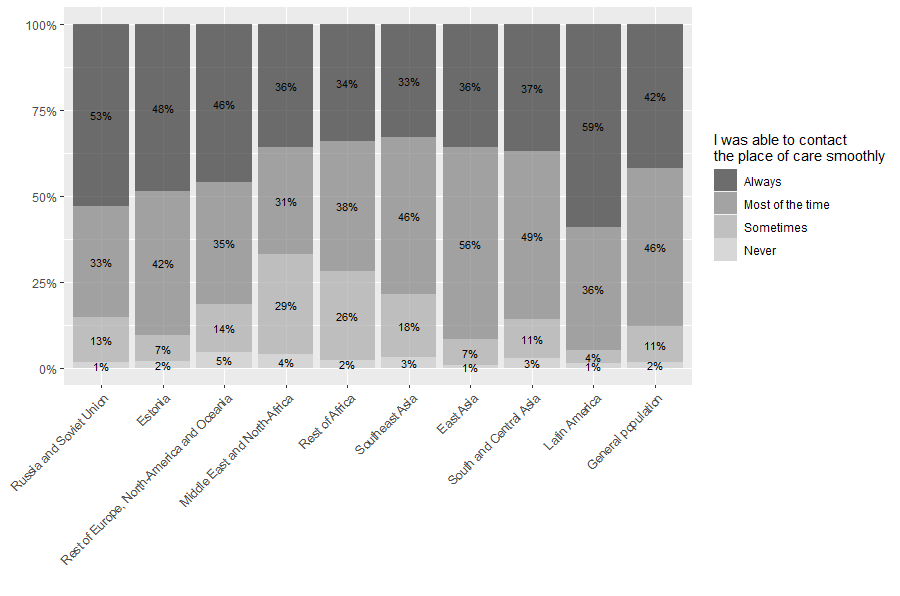


Supplementary figure 1. The distribution of responses to the item "I was able to contact the place of care smoothly" between migrants from different regions of origins and general population


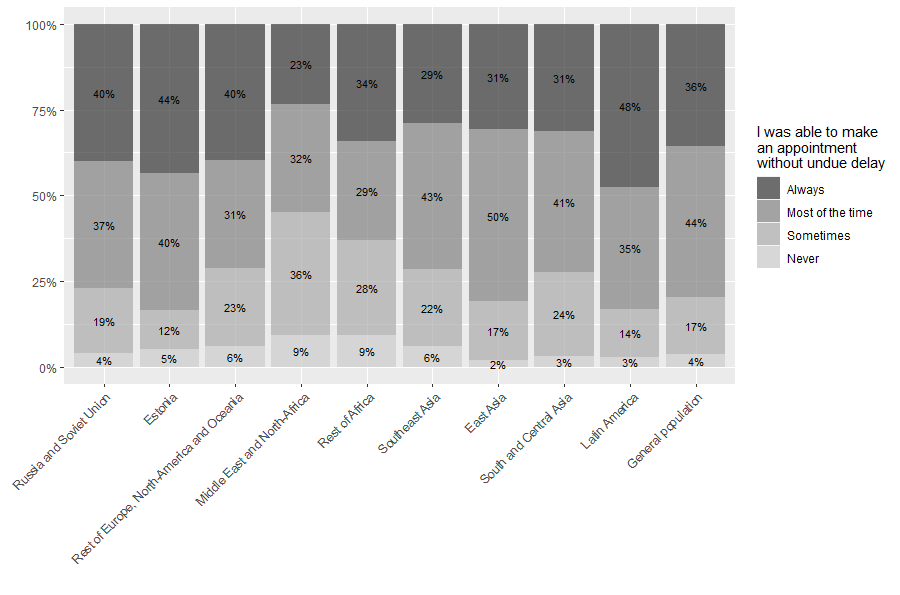


Supplementary figure 2. The distribution of responses to the item "I was able to make an appointment without undue delay" between migrants from different regions of origins and general population


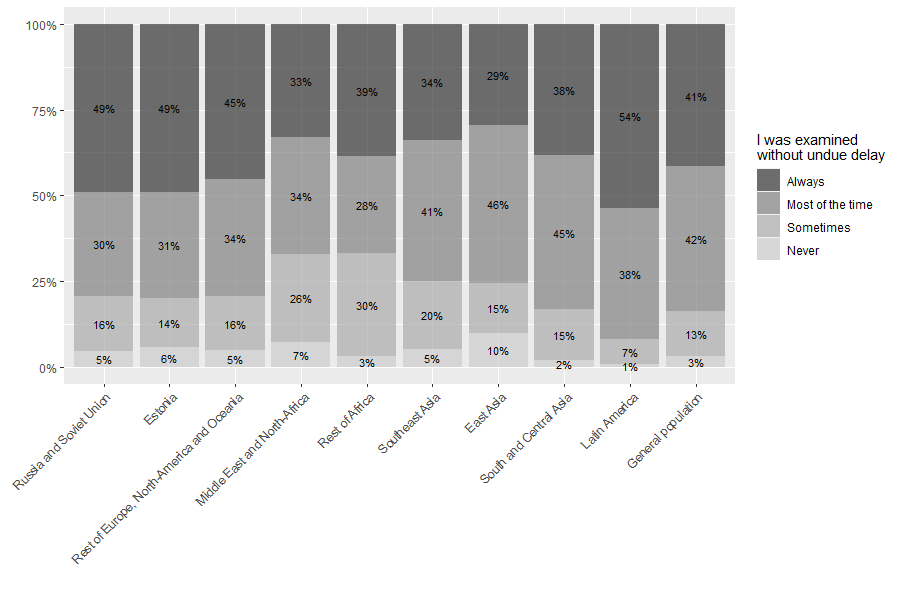


Supplementary figure 3. The distribution of responses to the item "I was examined without undue delay” between migrants from different regions of origin and general population.
